# Return to Work and Mortality in Breast Cancer Survivors: A 11-Year Longitudinal Study

**DOI:** 10.3390/ijerph192114418

**Published:** 2022-11-03

**Authors:** Zhe-Yu Yang, Wei-Liang Chen, Wei-Te Wu, Ching-Huang Lai, Ching-Liang Ho, Chung-Ching Wang

**Affiliations:** 1 Division of Family Medicine, Department of Family and Community Medicine, Tri-Service General Hospital, National Defense Medical Center, Taipei 114, Taiwan; 2 Division of Occupational Medicine, Department of Family & Community Medicine, Tri-Service General Hospital, National Defense Medical Center, Taipei 114, Taiwan; 3 National Institute of Environmental Health Science, National Health Research Institutes, Miaoli 350, Taiwan; 4 School of Public Health, National Defense Medical Center, Taipei 114, Taiwan; 5 Division of Hematology/Oncology, Department of Medicine, Tri-Service General Hospital, School of Medicine, National Defense Medical Center, Taipei 114, Taiwan

**Keywords:** breast cancer, return to work, survival outcome

## Abstract

Breast cancer is the most commonly occurring cancer in women, and it is a major cause of cancer death around the world. With the development of diagnostic methods and improvements in treatment methods, the incidence rate of breast cancer and the number of breast cancer survivors continue to simultaneously increase. We used national registry database to analyze the features that affect employment and return to work among breast cancer survivors. A total of 23,220 employees, who were newly diagnosed with breast cancer were recruited based on the Labor Insurance Database (LID), the Taiwan Cancer Registry (TCR), and National Health Insurance Research Database (NHIRD) during the period 2004–2015. The correlations between return to work (RTW) and independent confounding factors were examined using Cox proportional hazards model. Survival probability was analyzed using the Kaplan–Meir method. After adjusting for confounding variables, cancer stage, chemotherapy and higher income were significantly negatively correlated with RTW. Among breast cancer survivors, RTW was found to be related to a lower risk of all-cause mortality in both the unadjusted and fully adjusted model. Patients who had RTW exhibited better survival in all stages. Work-, disease- and treatment-related factors influenced RTW among employees with breast cancer. RTW was associated with better breast cancer survival. Our study demonstrates the impact of RTW and the associated factors on breast cancer survivorship.

## 1. Introduction

Breast cancer is the most commonly occurring cancer in women. Furthermore, it is a major cause of cancer death and has a high incidence rate around the world [1]. Studies show that the majority of white people with breast cancer are in their 60s, whereas the majority of nonwhite people with breast cancer are in their 40s in the US, meaning that they are categorized as being of mature working age [2]. Undoubtedly, this is a bigger issue as regards the US labor nonwhite force. Fortunately, improvements in breast cancer screening, such as mammography techniques, and treatments including less aggressive surgery, adjuvant chemotherapy, and hormonal therapy, have reduced the recurrence rates, mortality rates, and increased the survival rates in all patients with breast cancer [3,4,5]. According to the present study, breast cancer patients currently have a 5-year survival range from 79% to 93% in the European Union [6]. As the number of breast cancer survivors has increased, employment has become a big concern [7]. Breast cancer survivors face many challenges. There are both associated with the physical side effects of treatment, and with quality-of-life, psychological, social, and financial issues [8,9,10,11]. 

The ability to return to work (RTW) is a confirmation of the social or family status of cancer survivors and an indication that the cancer has been cured [12]. There are many studies discussing RTW in breast cancer survivors and the literature suggests that the majority of cancer survivors can return to work after treatment [13,14]. Previous studies showed multiple factors influencing RTW for breast cancer patients, such as socio-demographic characteristics, work-related factors, disease and treatment-related factors [13]. Moreover, the altitude of employers also had a pivotal role in successful return to work in breast cancer survivors [13]. Therefore, this is important to study the relationship between cancer survivors, RTW, and mortality.

In one study, 80% of breast cancer patients went on sick leave after diagnosis, but only 56% came back to work after finishing treatment [15]. In another review, researchers found that 43% to 93% of breast cancer patients could RTW within 1 year of diagnosis [16]. In a focus group study by Tamminga et al., breast cancer survivors who were 2 years post-diagnosis reported physical impairments related to the treatment as a barrier for their RTW [17]. Schmidt ME and her colleagues found that persistent tiredness and cognitive issues were correlated with no RTW [18]. Economic problems also play an important role among breast cancer survivors [9]. De Boer et al. reported that unemployment status is associated with cancer survivorship [19]. Lauzier et al. demonstrated wage losses and associated financial stress among breast cancer patients [20]. Drolet et al. showed that breast cancer patients took 6 months of sick leave on average [21]. Hence, being employed and RTW may lighten the financial burden and improve quality of life [22]. RTW also represents a return to normal life and leads to an improved social role [23].

To date, most studies exploring changes in employment status in cancer survivors have relatively short follow-up period [24]. There are few studies that focus on long-term employment status among breast cancer survivors [24]. Moreover, prior research mainly focused on Western populations [7]. This study’s objective was to analyze the effects of socio-economic factors, treatment factors, and disease-related factors among breast cancer survivors on RTW and mortality by conducting a national registry-based cohort study. We hope that this study highlights the benefits of returning to work for breast cancer survivors in Asian populations. 

## 2. Materials and Methods

### 2.1. Study Population 

We established a retrospective cohort study by recruiting employees based on the Labor Insurance Database (LID), the Taiwan Cancer Registry (TCR), and National Health Insurance Research Database (NHIRD) during the period 2004–2015 in Taiwan. Through links with the LID, TCR, and NHIRD using personal identification numbers, we obtained work-related data, including employment information, employee’s industry, monthly income, and company scale. Primary diagnosis of breast cancer is coded by the International Classification of Diseases for Oncology–3rd edition (ICD-O-3). In this cohort study, we enrolled 23,220 participants who were first diagnosed with breast cancer (ICD-O-3 C50) during the study period (2004–2010) and had a minimum follow-up time of 2 years from cancer diagnosis. The tracking period was from January 2004 to December 2015. We excluded participants who were younger than 20, who had two or more separate cancers, including breast cancer, and who were out of work at baseline. All protocols were executed by the Institutional Review Board (IRB) of Tri-Service General Hospital (TSGH).

### 2.2. Covariates 

Demographic data including age, employment information, employee’s industry, monthly income and company scale were obtained from the LID. We classified monthly income into USD ≤ 960, USD > 960~1273 and USD > 1273. We classified company scale into company closed, small (less than five people), medium (less than 200 people) and large (more than 200 people). Clinical comorbidities were collected from the NHIRD according to the International Classification of Diseases, 9th Revision, Clinical Modification codes. Clinical comorbidities included disorders of lipid metabolism, cerebrovascular diseases, chronic pulmonary diseases, peptic ulcer diseases, renal diseases, liver diseases, psychoses, and depression. The International Classification of Diseases, 9th Revision, Clinical Modification codes for clinical comorbidities mentioned above is listed in Supplementary Table S1. The pathological breast cancer stage and treatment types for breast cancer, such as surgical management, radiation therapy, and chemotherapy were also collected from the NHIRD. Covariates were assessed at the time of 2 years after cancer diagnosis.

### 2.3. Outcome Assessment 

The primary outcome of our study was RTW 2 years after breast cancer diagnosis. RTW was selected as the primary outcome because RTW of breast cancer patients was regarded as an important part of recovery. Every enrolled worker was followed from the date of primary diagnosis of breast cancer to the end of the follow-up or to death. The secondary outcome was the all-cause mortality within the follow-up period.

### 2.4. Statistical Analysis 

We utilized the SAS (software) package (SAS 9.3, SAS Institute Inc., Cary, North Carolina) to perform our data analyses. Two-sided *p* values < 0.05 were considered to be statistically significant. Continuous variables are indicated as the means and standard deviations (SD); categorical variables are indicated as frequencies and percentages. The chi-squared test was used for testing relationships between categorical variables and the Wilcoxon rank-sum test or independent *t*-test was used for continuous variables. Employment status or RTW was evaluated 2 years after breast cancer diagnosis. Survival time was followed from the first breast cancer diagnosis to the date of death between 1 January 2004 and 31 December 2015. Univariate and multivariate analyses using Cox proportional hazard models were performed to assess the effect of covariates on RTW. The correlation between RTW and all-cause mortality among breast cancer patients was also investigated by the multivariate Cox proportional hazards model. Age, gender, pathological breast cancer stage, received treatment, monthly income, employee’s industry, and company scale were included in the fully adjusted model. Survival probability was analyzed using the Kaplan–Meir method.

## 3. Results

### 3.1. Characteristics of the Study Population

A total of 16,083 newly diagnosed female breast cancer patients were enrolled and analyzed in the study. The mean age of the participants was 48.2 ± 7.8 years, and more than half of patients (62.8%) had a monthly income range below USD 960. From all the cases, 5880 were stage 2 (36.5%), followed by stage 1 (32.7%), stage 3 (15.6%), stage 0 (13%) and stage 4 (2%). In addition, 93.9% of women underwent surgical treatment and 42.7% received chemotherapy. The main industry category was manufacturing (31.7%). Other covariates, including comorbidities before cancer diagnosis and company scale, are listed in Table 1. We divided the study population into an RTW group and non-RTW group. The RTW group included patients who were continuing employment and those who were re-employed. The non-RTW group included patients who were unemployed or did not RTW. In general, the data show that the non-RTW group had a higher prevalence of suffering comorbidities and chemotherapy treatment, had a higher income, worked in small- to medium-sized companies, and exhibited a higher pathology stage.

### 3.2. Univariate Analysis of Independent Factors Associated with RTW in Cox Proportional Hazards Models

Table 2 presents the factors associated with RTW in the 2-year univariate analysis using Cox proportional hazards models. Early-stage cancer was related to a higher likelihood of RTW. On the contrary, factors including age, receiving chemotherapy or hormone replacement therapy, a monthly income over USD 1273, working in small- to medium-sized company were associated with a lower likelihood of RTW. Several industry categories, including the wholesale and retail trade (wholesale), information and communication (information), financial and insurance activities (financial activity), professional, scientific and technical activities (technical activates), public administration and defense (public administration) and human health and social work activities (human health) were correlated with a lower likelihood of RTW.

### 3.3. Multivariate Analysis of Independent Factors Associated with RTW in Cox Proportional Hazards Models

Table 3 presents variables associated with RTW in the 2-year multivariate analysis using Cox proportional hazards models. After adjusting for other variables, Table 3 shows almost the same outcomes as Table 2, except for the industry categories. The association between industry categories and RTW was not statistically significant after adjusting for confounding factors. Cancer stage was inversely associated with likelihood of RTW. Patients who received chemotherapy were less likely to RTW. Patients with a higher income (a monthly income above USD1273) were less likely to RTW. Patients working in small- to medium-sized company were less likely to RTW.

### 3.4. Influence of RTW on Survival Outcomes

**Figure 1:**
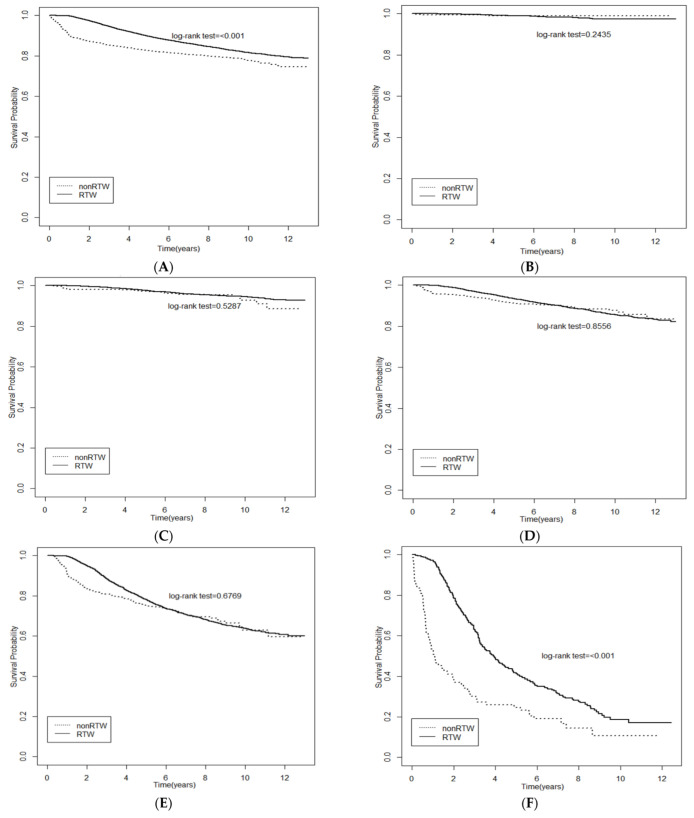
Kaplan-Meier (KM) curves showed survival probability of RTW for all (**A**) and stage 0–4 (**B**–**F**) breast cancer employers. (**A**) All stage; (**B**) Stage 0; (**C**) Stage 1; (**D**) Stage 2; (**E**) Stage 3; (**F**) Stage 4.

Figure 1 shows the significant differences of survival probability between the RTW group and non-RTW group using Kaplan–Meier curves. A better survival probability was found in the RTW group than the non-RTW group in participants with stage 2 (Figure 1D), stage 3 (Figure 1E), and stage 4 cancer (Figure 1F). Table 4 presents the relationship between the independent variates and all-cause mortality in the multivariate Cox proportional hazard models. RTW was found to be related to a lower risk of all-cause mortality among breast cancer patients in the fully adjusted model (HR = 0.585; 95% CI, 0.526 to 0.649).

## 4. Discussion

We conducted a retrospective cohort study to evaluate the factors that affect employment status in newly diagnosed female breast cancer patients. We analyzed 23,220 newly diagnosed female breast cancer patients from 2004 to 2010 and tracked whether these patients returned to work or not from 2004 to 2015. We demonstrated that early-stage breast cancer was positively associated with RTW, and higher income and receiving chemotherapy were negatively correlated with RTW. RTW was also found to be associated with better survival among breast cancer patients.

Multiple factors were identified in previous research related to RTW, such as education, ethnicity, chemotherapy, heavy physical work, poor health, fatigue, depression and emotional distress [16]; however, few studies include factors such as comorbidities, industry category, with a follow-up of more than 5 years in Asian populations [25,26]. The results of these studies showed that socio-demographic, disease-related, and work-related factors are important elements in RTW. Socio-demographic factors, for the example patient’s age, educational level, ethnicity, and marital status, were correlated with RTW [25,27]. A retrospective cohort study from Canada noted that older breast cancer patients were less likely to return to work [27]. A low educational level was found to be associated with unemployment [14]. Due to limitations related to our database, age was the only socio-demographic factor highlighted in this study. Age was significantly associated as a negative factor with RTW in the univariate (HR = 0.996; 95% CI, 0.994~0.998) and multivariate (HR = 0.995; 95% CI, 0.993~0.997) regression models. Psychological factors, including worry, depression, and frustration were found to affect RTW [28]. An observational study by Wolvers et al. revealed self-efficacy was correlated with earlier RTW [29]. 

Disease-related factors, such as stage of breast cancer, surgery, chemotherapy, radiotherapy, post-treatment side effects (fatigue, pain, nausea, vomiting, arm morbidity, and cognitive dysfunction), and multiple co-morbidities were reported to affect RTW [13,25,30,31]. Fantoni et al. showed that chemotherapy and radiotherapy were correlated with both limited and delayed RTW [31]. In a health insurance database study, the results demonstrated that chemotherapy was related with long-term disability, stopping working, and retirement in female breast cancer patients [32]. Our study was consistent with these studies. The cancer stage was another important factor that was found to be inversely related with work status and employment in previous studies [25,33]. In a French prospective study, advanced stage was correlated with unemployment [34]. A similar pattern was noted in our study. 

Work-related factors, such as job type, workplace accommodation or discrimination and average salary were associated with RTW [13,25,35]. Although there are few work-related factors, such as industry category, income, and the company size in our database, we found different industry categories with different patterns of RTW in the univariate analysis. For example, industries including wholesale and retail trade (wholesale), financial and insurance activities (financial activity), scientific and technical activities (technical activates) and public administration and defense (public administration) were associated with a lower likelihood of RTW as follow-up years increased. On the other hands, industries such as professional, information and communication (information) and human health and social work activities (human health) were correlated with a higher likelihood of RTW as follow-up years increased. The results may be similar to those reported in prior research regarding manual work [36] and heavy lifting being a barrier to RTW [16]. 

Interestingly, higher income and work in small- or medium-sized company had a lower rate of RTW compared with a lower income and large-sized company in our study. Previous studies demonstrated that a low income was correlated with unemployment, as compared with a higher income [25,27,36,37]. Moreover, employers’ understanding of breast cancer diagnosis, treatment, and recovery is related to RTW [13]. Another cohort of 131 cancer survivors reported that socio-demographic, health-, and work-related factors influenced RTW, including company size [38]. This inconsistency may be due to the limitations of our database, including the lack of workplace accommodation, discrimination or work adjustments data, or important factors obscured by company size.

Prognostic or survival factors for breast cancer including age, cancer stage, tumor receptor status, tumor size, lymph node involvement, and histologic type, are well established [39,40]. Studies examining a variety of comorbidity related risk factors associated with breast cancer survival have been published in recent years. Obesity was correlated with poorer survival among breast cancer patients [41]. Diabetes mellitus was related with a higher risk of mortality in breast cancer survivors [42]. In addition to the common risk factors mentioned above, our study also found that RTW was negatively associated with all-cause mortality (HR = 0.585; 95% CI, 0.526 to 0.649) in a fully adjusted model. This finding should be studied more carefully to explore the possible factors related to the disease, treatment, and the workplace.

Taiwanese National Health Insurance has a coverage rate of nearly 99.9% [43]. Comprehensive content including cancer treatment costs, is provided by the Taiwanese National Health Insurance system [44]. Moreover, the Taiwan labor insurance offers 50% of the average daily insurance salary for compensation of lost wages during hospitalization. This may account for the major difference in social welfare between breast cancer survivors from Taiwan and in other countries. Cultural differences including self-stigma and gender stereotyping might affect willingness to RTW for Asian cancer patients [45,46]. However, as a result of the limitations of our database, future studies are required to address these issues.

There are several limitations in our study. Firstly, there was a lack of information about socio-demographic and work-related factor, such as family support, educational level, ethnicity, marital status, work accommodation, and discrimination, which may represent confounding factors affecting RTW, in the study database. Second, there was no available data regarding quality of life. We did not access the relationship between quality of life and RTW among breast cancer survivors. Third, the generalizability of our findings is limited because (1) our study participants were only recruited from Taiwan and (2) this study was conducted in Taiwan, which has a distinctive public welfare system.

## 5. Conclusions

The present study demonstrated that RTW was associated with better breast cancer survival. Cancer stage was inversely correlated with likelihood of RTW. Chemotherapy and working in a small- or medium-sized company had a negative effect on RTW. The results revealed the impact of RTW and associated factors on breast cancer survivorship. Further research is required to access other confounding variables, such as work accommodation, work discrimination, quality of life and social support.

## Figures and Tables

**Table 1:** Characteristics of the study population.

Characteristic	All	RTW	%	Non-RTW	%
**Age (year) mean ± SD (range)**	48.2 ± 7.8 (19–86)	47.9 ± 7.6 (19–86)		49.2 ± 8.6 (19–84)	
**Age group**					
<40	2107	1726	13.1	381	12.7
40–49	6826	5668	43.2	1158	38.7
50–59	6170	5049	38.5	1121	37.5
60–69	916	609	4.6	307	10.2
≥70	64	44	0.3	20	0.6
**Comorbidities**					
Disorders of lipoid metabolism	1272	1007	7.6	265	8.8
Obesity	45	36	0.2	9	0.3
Hypertension	2129	1686	12.8	443	14.8
Congestive heart failure	104	84	0.6	20	0.6
Peripheral vascular disease	91	74	0.5	17	0.5
Cerebrovascular disease	170	139	1	31	1
Chronic pulmonary disease	485	390	2.9	95	3.1
Rheumatologic disease	202	170	1.2	32	1
Peptic ulcer disease	854	685	5.2	169	5.6
Mild liver disease	822	671	5.1	151	5
Hemiplegia or paraplegia	13	10	0.07	3	0.1
Renal disease	129	105	0.8	24	0.8
Psychoses	71	55	0.4	16	0.5
Depression	433	354	2.7	79	2.6
**Comorbidity**					
0	11,423	9351	71.4	2072	69.3
1	2837	2295	17.5	542	18.1
2	1307	1036	7.9	271	9
≥3	516	414	3.1	102	3.4
**Treatment**					
OP	15,108	12,321	94	2787	93.3
RTB	7011	5687	43.4	1324	44.3
CH	6881	5390	41.1	1491	49.9
HORM	6937	5453	41.6	1484	49.6
**Monthly income (USD)**					
≤960	10,110	8851	67.5	1259	42.1
>960~1273	2735	2307	17.6	428	14.3
>1273	3238	1938	14.7	1300	43.5
**Employee’s industry**					
Agriculture	794	705	5.3	89	2.9
Manufacturing	5112	4236	32.3	876	29.3
Electricity and Gas Supply	19	12	0.09	7	0.2
Water Supply	72	60	0.4	12	0.4
Construction	1063	918	7	145	4.8
Wholesale and Retail Trade	2175	1663	12.6	512	17.1
Transportation and Storage	672	549	4.1	123	4.1
Accommodation	737	623	4.7	114	3.8
Information and Communication	277	201	1.5	76	2.5
Financial and Insurance Activities	758	568	4.3	190	6.3
Real Estate Activities	190	165	1.2	25	0.8
Technical Activities	531	389	2.9	142	4.7
Support Service Activities	403	322	2.4	81	2.7
Public Administration	385	283	2.1	102	3.4
Education	364	290	2.2	74	2.4
Human Health and Social Work Activities	652	486	3.7	166	5.5
Arts, Entertainment and Recreation	198	176	1.3	22	0.7
Other Service Activities	1681	1450	11	231	7.7
**Company scale**					
Company closed	1430	1122	8.5	308	10.3
Small	1308	1003	7.6	305	10.2
Medium	3676	2797	21.3	879	29.4
Large	9669	8174	62.4	1495	50
**Pathological stage**					
0	2094	1711	13	383	12.8
1	5270	4350	33.2	920	30.8
2	5880	4867	37.1	1013	33.9
3	2515	1958	14.9	557	18.6
4	324	210	1.6	114	3.8

Abbreviation: SD = standard deviation, OP = operation, RTB = radiotherapy, CH = chemotherapy, HORM = hormone therapy.

**Table 2:** Univariate analysis of RTW in Cox proportional hazards models.

Characteristic	2-Year RTW	
	HR (95% CI)	*p* Value
**Age (year) mean ± SD (range)**	0.996 (0.994–0.998)	0.0004
**Age group**		
<40	Reference	
40–49	1.04 (0.985–1.098)	0.1535
50–59	1.027 (0.972–1.085)	0.337
60–69	0.782 (0.713–0.858)	<0.0001
≥70	0.821 (0.609–1.107)	0.1966
**Comorbidities (Reference: no)**		
Disorders of lipoid metabolism	0.965 (0.905–1.029)	0.2811
Obesity	0.973 (0.701–1.349)	0.868
Hypertension	0.966 (0.918–1.017)	0.1847
Congestive heart failure	1 (0.807–1.239)	1
Peripheral vascular disease	1.009 (0.803–1.268)	0.9369
Cerebrovascular disease	1.01 (0.855–1.194)	0.9065
Chronic pulmonary disease	0.988 (0.893–1.093)	0.8114
Rheumatologic disease	1.055 (0.907–1.227)	0.4862
Peptic ulcer disease	0.981 (0.909–1.06)	0.6331
Mild liver disease	1.007 (0.932–1.088)	0.858
Hemiplegia or paraplegia	0.949 (0.511–1.764)	0.8693
Renal disease	1.016 (0.839–1.231)	0.8704
Psychoses	0.996 (0.878–1.129)	0.9467
Depression	0.941 (0.722–1.226)	0.6542
**Comorbidity**		
0	Reference	
1	0.991 (0.946–1.037)	0.6858
2	0.964 (0.904–1.028)	0.2644
≥3	0.985 (0.892–1.086)	0.7572
**Treatment (Reference: no)**		
OP	1.032 (0.96–1.11)	0.3934
RTB	0.987 (0.954–1.022)	0.4682
CH	0.91 (0.879–0.942)	<0.0001
HORM	0.917 (0.886–0.95)	<0.0001
**Monthly income (USD)**		
≤960	Reference	
>960~1273	0.952 (0.91–0.997)	0.0369
>1273	0.595 (0.566–0.625)	<0.0001
**Employee’s industry**		
Agriculture	0.999 (0.847–1.178)	0.9897
Manufacturing	0.894 (0.769–1.04)	0.1454
Electricity and Gas Supply	0.634 (0.354–1.138)	0.127
Water Supply	0.881 (0.657–1.181)	0.3964
Construction	0.955 (0.813–1.122)	0.5747
Wholesale and Retail Trade	0.79 (0.676–0.923)	0.003
Transportation and Storage	0.881 (0.743–1.044)	0.1426
Accommodation	0.92 (0.779–1.088)	0.3314
Information and Communication	0.729 (0.596–0.893)	0.0022
Financial and Insurance Activities	0.775 (0.655–0.918)	0.0032
Real Estate Activities	0.937 (0.757–1.158)	0.5452
Technical Activities	0.745 (0.624–0.891)	0.0012
Support Service Activities	0.842 (0.701–1.012)	0.0667
Public Administration	0.754 (0.625–0.911)	0.0033
Education	0.811 (0.672–0.978)	0.0283
Human Health and Social Work Activities	0.761 (0.64–0.904)	0.0019
Arts, Entertainment and Recreation	Reference	
Other Service Activities	0.955 (0.817–1.117)	0.5678
**Company scale**		
Company closed	0.869 (0.816–0.925)	<0.0001
Small	0.857 (0.802–0.915)	<0.0001
Medium	0.846 (0.811–0.884)	<0.0001
Large	Reference	
**Pathological stage**		
0	1.338 (1.159–1.544)	<0.0001
1	1.357 (1.182–1.559)	<0.0001
2	1.36 (1.185–1.562)	<0.0001
3	1.259 (1.092–1.452)	0.0015
4	Reference	

Abbreviation: HR = hazard ratio, CI = confidence interval, RTW = return to work, SD = standard deviation, OP = operation, RTB = radiotherapy, CH = chemotherapy, HORM = hormone therapy.

**Table 3:** Multivariate analysis of RTW in Cox proportional hazards models.

Characteristics	2-Year RTW	
	HR (95% CI)	*p* Value
**Age (year) mean ± SD (range)**	0.995 (0.993–0.997)	<0.0001
**Age group**		
<40	Reference	
40-49	1.027 (0.973–1.085)	0.3347
50-59	1.003 (0.947–1.062)	0.9261
60-69	0.773 (0.702–0.851)	<0.0001
≥70	0.744 (0.55–1.006)	0.0543
**Comorbidity**		
0	Reference	
1	0.991 (0.946–1.038)	0.6998
2	0.973 (0.911–1.039)	0.4177
≥3	1.005 (0.909–1.111)	0.9211
**Treatment (Reference: no)**		
OP	1.032 (0.959–1.11)	0.4052
RTB	1.015 (0.979–1.052)	0.4107
CH	0.926 (0.887–0.965)	0.0003
HORM	0.957 (0.92–0.996)	0.0296
**Monthly income (USD)**		
≤960	Reference	
>960~1273	0.985 (0.94–1.032)	0.5237
>1273	0.612 (0.58–0.646)	<0.0001
**Employee’s industry**		
Agriculture	0.937 (0.794–1.106)	0.4435
Manufacturing	0.919 (0.79–1.069)	0.2743
Electricity and Gas Supply	0.814 (0.453–1.462)	0.4905
Water Supply	0.929 (0.693–1.246)	0.6244
Construction	0.956 (0.813–1.123)	0.5816
Wholesale and Retail Trade	0.904 (0.772–1.059)	0.212
Transportation and Storage	0.922 (0.778–1.093)	0.3523
Accommodation	0.895 (0.757–1.058)	0.1943
Information and Communication	0.859 (0.701–1.053)	0.1429
Financial and Insurance Activities	0.99 (0.834–1.175)	0.9068
Real Estate Activities	0.999 (0.808–1.236)	0.994
Technical Activities	0.865 (0.723–1.036)	0.1145
Support Service Activities	0.868 (0.722–1.043)	0.1315
Public Administration	0.854 (0.706–1.033)	0.1035
Education	0.852 (0.705–1.029)	0.0963
Human Health and Social Work Activities	0.853 (0.717–1.015)	0.0723
Arts, Entertainment and Recreation	Reference	
Other Service Activities	0.943 (0.807–1.103)	0.4663
**Company scale**		
Company closed	0.898 (0.841–0.96)	0.0015
Small	0.877 (0.819–0.941)	0.0002
Medium	0.888 (0.848–0.931)	<0.0001
Large	Reference	
**Pathological stage**		
0	1.368 (1.182–1.584)	<0.0001
1	1.405 (1.222–1.616)	<0.0001
2	1.384 (1.204–1.589)	<0.0001
3	1.261 (1.093–1.455)	0.0015
4	Reference	

Abbreviation: HR = hazard ratio, CI = confidence interval, RTW = return to work, OP = operation, RTB = radiotherapy, CH = chemotherapy, HORM = hormone therapy.

**Table 4:** Multivariate analysis of relationship between independent variables and all-cause mortality of breast cancer patients.

Characteristic	Fully Adjusted HR (95% CI)	*p* Value
**RTW (Ref: non-RTW)**	0.585 (0.526–0.649)	<0.0001
**Age (Ref: age <40)**		
40–49	0.735 (0.643–0.84)	<0.0001
50–59	0.901 (0.789–1.029)	0.1245
60–69	0.949 (0.744–1.164)	0.6154
≥70	2.232 (1.444–3.451)	0.0003
**Treatment (Ref: no)**		
OP	0.698 (0.603–0.809)	<0.0001
RTB	0.822 (0.75–0.901)	<0.0001
CH	1.013 (0.908–1.13)	0.8179
HORM	0.692 (0.617–0.775)	<0.0001
**Monthly income (Ref: ≤$960)**		
>960~1273	0.879 (0.775–0.995)	0.0418
>1273	0.71 (0.62–0.812)	<0.0001
**Pathological stage (Ref: stage 4)**		
0	0.014 (0.01–0.019)	<0.0001
1	0.037 (0.031–0.045)	<0.0001
2	0.089 (0.076–0.103)	<0.0001
3	0.283 (0.244–0.329)	<0.0001

Abbreviation: HR = hazard ratio, RTW = return to work. Adjusted covariates: age, gender, pathological stage of lung cancer, received treatment, monthly income, employee’s industry and company size.

## Data Availability

The data underlying this study are from the Labor Insurance Database (LID) and National Health Insurance Research Database (NHIRD). The LID and NHIRD is not free to public access, and therefore, interested researchers can obtain the data through formal application to the Health and Welfare Data Science Center, Ministry of Health and Welfare, Taiwan. (https://dep.mohw.gov.tw/DOS/np-2497-113.html). Last accessed date: 29 June 2020.

## Supplementary Materials

The following are available online at https://www.mdpi.com/article/10.3390/ijerph192114418/s1, Table S1: The ICD-9-CM codes for clinical comorbidities.
