# Supplementary material for: Return to Work and Mortality in Breast Cancer Survivors: A 11-Year Longitudinal Study
Source: Int J Environ Res Public Health. 2022 Nov 3;19(21):14418. doi: 10.3390/ijerph192114418 (PMC9655987; doi:10.3390/ijerph192114418)
Supplement: Supplementary file 1 [file ijerph-19-14418-s001.zip › ijerph-1941175-supplementary.pdf]

Supplemental Table S1: The ICD-9-CM codes for clinical comorbidities

Comorbidities	ICD-9-CM codes
Disorders of lipid metabolism	ICD-9-CM codes, 272
Cerebrovascular diseases	ICD-9-CM codes, 362.34, 430.x-438.x
Chronic pulmonary diseases	ICD-9-CM codes, 416.8, 416.9, 490.x-505.x, 506.4, 508.1, 508.8
Peptic ulcer diseases	ICD-9-CM codes, 531.x-534.x
Renal diseases	ICD-9-CM codes, 403.01, 403.11, 403.91, 404.02, 404.03, 404.12, 404.13, 404.92, 404.93, 582.x, 583.0-583.7, 585.x, 586.x, V42.0, V45.1, V56.x
Liver diseases	ICD-9-CM codes, 070.22, 070.23, 070.32, 070.33, 070.44, 070.54, 070.6, 070.9, 570.x, 571.x, 573.3, 573.4, 573.8, 573.9, V42.7
Depression	ICD-9-CM codes, 296.2, 296.3, 296.5, 300.4, 309.x, 311.x
